# Detection and dissemination of *Toxoplasma gondii* in experimentally infected calves, a single test does not tell the whole story

**DOI:** 10.1186/s13071-018-2632-z

**Published:** 2018-01-18

**Authors:** Alison Burrells, Alessandra Taroda, Marieke Opsteegh, Gereon Schares, Julio Benavides, Cecile Dam-Deisz, Paul M. Bartley, Francesca Chianini, Isabella Villena, Joke van der Giessen, Elisabeth A. Innes, Frank Katzer

**Affiliations:** 1 Moredun Research Institute, Pentlands Science Park, Bush Loan, Penicuik, Scotland; 2 Protozoology Laboratory, Departamento de Medicina Veterinária Preventiva, Universidade Estadual de Londrina – UEL, Londrina, PR Brazil; 3 National Institute for Public Health and the Environment (RIVM), Centre for Infectious Disease Control, Bilthoven, The Netherlands; 4 Friedrich-Loeffler-Institut, Bundesforschungsinstitut für Tiergesundheit, Federal Research Institute for Animal Health, - Insel Riems, Greifswald, Germany; 5 Instituto de Ganadería de Montaña (CSIC-ULE), León, Spain; 6 Laboratoire de Parasitologie-Mycologie, Centre National de Référence de la Toxoplasmose, Centre de Ressources Biologiques Toxoplasma, Cédex, Reims, France

**Keywords:** Cattle, *Toxoplasma gondii*, Food safety, Beef, Mouse bioassay, Magnetic capture, Polymerase chain reaction

## Abstract

**Background:**

Although the detection of *Toxoplasma gondii* in bovine tissues is rare, beef might be an important source of human infection. The use of molecular techniques, such as magnetic capture qPCR (MC-qPCR), in combination with the gold standard method for isolating the parasite (mouse bioassay), may increase the sensitivity of *T. gondii* detection in infected cattle. The risk of transmission of the parasite to humans from undercooked/raw beef is not fully known and further knowledge about the predilection sites of *T. gondii* within cattle is needed. In the current study, six Holstein Friesian calves (*Bos taurus*) were experimentally infected with 10^6^ *T. gondii* oocysts of the M4 strain and, following euthanasia (42 dpi), pooled tissues were tested for presence of the parasite by mouse bioassay and MC-qPCR.

**Results:**

*Toxoplasma gondii* was detected by both MC-qPCR and mouse bioassay from distinct pools (100 g) of tissues comprising: liver, tongue, heart, diaphragm, *semitendinosus* (hindlimb), *longissimus dorsi* muscle (sirloin) and *psoas major* muscle (fillet). When a selection of individual tissues which had been used for mouse bioassay were examined by MC-qPCR, parasite DNA could only be detected from two animals, despite all calves showing seroconversion after infection.

**Conclusions:**

It is apparent that one individual test will not provide an answer as to whether a calf harbours *T. gondii* tissue cysts. Although the calves received a known number of infectious oocysts and highly sensitive methods for the detection of the parasite within bovine tissues were applied (mouse bioassay and MC-qPCR), the results confirm previous studies which report low presence of viable *T. gondii* in cattle and no clear predilection site within bovine tissues.

## Background

Unlike sheep, natural infection of *T. gondii* in cattle does not appear to give rise to clinical signs or abortion in pregnant cows [1]. However, ingestion of *T. gondii* tissue cysts from infected meat is a major route of infection for humans, with consumption of raw or undercooked meat from infected animals considered a significant public health risk [2]. In the Netherlands, a quantitative microbial risk assessment (QMRA) model identified under-cooked/raw beef as potentially the most important source of *Toxoplasma* infection for the Dutch population [3].

The reported seroprevalence for *T. gondii* infection in cattle varies between different countries, for example 83.3% (*n* = 504) in southern Spain [4], 45.6% (*n* = 406) in Switzerland [5], 33.8% (*n* = 74) in eastern Poland [6], and 25.0% (*n* = 995) in cattle over 12 months old from the Netherlands [7]. In contrast to these figures, a much lower seroprevalence of 7.5% (*n* = 161) was reported for cattle from North Portugal [8]. Despite the high seroprevalence observed in the majority of studies, *T. gondii* parasites are rarely detected in seropositive animals.

Research completed by Opsteegh et al. [7] found that seroprevalence and isolation of *T. gondii* DNA from cattle tissues showed conflicting results, whereby 3% (2/66) of cattle which tested negative by ELISA and MAT actually tested positive by MC-qPCR (using 100 g heart tissue), whereas no parasite DNA was detected in seropositive animals. This result indicates that in this particular study, despite the animals being seronegative, *T. gondii* DNA was present within heart tissues of two animals. This result provides an indication that, in cattle, the detection of antibodies may not be directly linked to the presence of *T. gondii* DNA. It also raises the question as to whether detection of *T. gondii* DNA reflects the presence of infectious parasites (e.g. tissue cysts), since detection of viable infective *T. gondii* tissue cysts from cattle which have been either infected experimentally or naturally is rare [9, 10]. The use of improved more sensitive techniques for the molecular detection of parasite DNA (such as MC-qPCR), in conjunction with mouse bioassay, could provide new information on the dissemination, presence and viability of *T. gondii* within cattle.

The main objectives of this research were to identify the predilection sites of *T. gondii* in cattle, which may influence future sampling and, from a public health perspective, examine dissemination of the parasite to edible tissues. In addition, the relationship between the presence of antibodies against *T. gondii* and direct detection of infective *T. gondii* or *T. gondii* DNA in meat and other edible tissues was examined. Overall, the results can aid the selection of sampling sites for epidemiological studies and provide a better understanding of the risk of *T. gondii* infected beef for human consumption.

## Methods

### Cattle

To establish a *T. gondii* infection in cattle, six Holstein Friesian calves (*Bos taurus*), aged 6 weeks (serologically negative for *T. gondii* by MAT), were each orally infected with 1 × 10^6^ *T. gondii* oocysts of the M4 strain (day 0). All calves were housed together at Moredun Research Institute throughout the experiment and after weaning fed a commercial calf feed with water available ad libitum.

Rectal temperatures of all calves were monitored daily for 18 days post-infection (pi). Blood sampling was carried out weekly (days 0 to 42 pi) by jugular venepuncture into 10 ml vacutainer serum tubes. Blood was left to clot overnight at 4 °C then centrifuged at 2000×*g* for 10 min. Serum was transferred to sterile 1.5 ml tubes and stored at -20 °C. One calf (109) had to be excluded due to severe non-responsive pneumonia (unrelated to *T. gondii* infection) prior to the end of the experiment (day 40 pi) and could not be included in the mouse bioassay and pooled testing by MC-qPCR. However, data from rectal temperature and serological analysis were recorded and individual tissue samples were tested by MC-qPCR. All remaining calves were euthanised 42 days pi. Post-mortem examination and collection of 50 g tissue samples (brain, heart, diaphragm, masseter, tongue, liver, *musculus psoas major* (fillet), *musculus longissimus dorsi* (sirloin), left *musculus triceps femoralis* (forelimb) and left *musculus semitendinosus* (hindlimb), was carried out immediately. Tissue pools were prepared with samples from two or three calves per pool, and tested by bioassay and MC-qPCR.

### MAT

Serum samples from calves were collected weekly throughout the experiment and tested for the presence of *T. gondii* antibodies at the Laboratory of Parasitology-Mycology, Reims, France, using the modified agglutination test (MAT) as described in [11]. A sample was deemed positive if the antibody titre was equal to or greater than 1:6.

### Mouse bioassay

Forty Swiss Webster mice were used for the bioassay of calf tissue pools. Mice were monitored twice daily with food and water supplied ad libitum. Mice were divided into ten separate groups, with 2 mice inoculated with the homogenate of a pool of tissue from 2 to 3 calves. A total of 100 g of each specific pool was prepared with tissue from 2 or 3 calves (50 g or 33.3 g per tissue per calf). There were 10 pools in total, one per each location sampled: brain, heart, diaphragm, masseter, tongue, liver, *musculus psoas major* (fillet), *musculus longissimus dorsi* (sirloin), left *musculus triceps femoralis* (forelimb) and left *musculus semitendinosus* (hindlimb).

A trypsin digest was performed on each tissue pool and the subsequent homogenate for inoculation prepared with antibiotics as described in [12]. Each homogenate (approximately 5 ml) was stored at 4 °C overnight before two mice were both inoculated with 1 ml of homogenate via the intraperitoneal route. The remaining homogenate (approximately 3 ml) was stored at -20 °C for subsequent DNA extraction. Mice were culled by cervical dislocation when they showed signs of toxoplasmosis, or at the end of the six week bioassay. At post-mortem, blood samples were collected for antibody detection of the parasite and brain tissue collected for DNA extraction and *T. gondii* specific 529 bp qPCR. Brain smears were not made for microscopic examination of the parasite.

### DNA extraction from mouse brain and tissue homogenate

The Nucleospin Tissue kit from Macherey-Nagel (Fisher Scientific, Loughborough, UK) was used to extract genomic DNA from both mouse brains and tissue homogenate. For mouse brains, each brain was homogenised in 1 ml of sterile PBS by passing through an 18G needle several times. The Macherey-Nagel standard protocol for human or animal tissue and cultured cells protocol was then followed using 100 μl of homogenised mouse brain. For the remaining calf tissue homogenate, 1440 μl of buffer T1 and 200 μl of proteinase K were added to 200 μl of tissue homogenate. Following incubation at 65 °C for 1 h, 230 μl was removed and processed following the Macherey-Nagel standard protocol for human or animal tissue and cultured cells from “step 3” onwards. All extracted DNA samples were stored at -20 °C prior to analysis. A DNA extraction control was included each time the method was completed.

### ELISA for mouse serum examination

All mouse sera were tested by ELISA for *T. gondii* IgG (ID.Vet, Montpellier, France). The ELISA was completed as described by the manufacturer’s instructions and as reported in [13]. In addition, confirmatory tests were performed using immunoblots of total tachyzoite *T. gondii* antigen or purified p30 (TgSAG1) antigen as described below.

### Immunoblot for mouse serum examination

For Western blot, total cell culture derived tachyzoites or purified surface antigens of *T. gondii* RH were used as described in [14], but with few modifications. For immunoblot, either pellets of 2 × 10^8^ tachyzoites or purified p30 (0.05 μg) were incubated in non-reducing sample buffer [2% (*w*/*v*) sodium dodecyl sulfate (SDS), 10% (*v*/v) glycerol, 62 mM Tris-HCl, pH 6.8] for 1 min (94 °C), separated in 12% (w/v) SDS polyacrylamide minigels (60 × 70 × 1 mm) and transferred to PVDF membranes (Immobilon-P, Millipore, Darmstadt, Germany). After the transfer, membranes were blocked using PBS-TG [PBS with 0.05% (v/v) Tween 20 (Sigma, Darmstadt, Germany) and 2% (v/v) liquid fish gelatine (Serva, Heidelberg, Germany)] and cut into 50 strips and examined as described below. Sera were diluted 1:20 in PBS-TG. To detect antibodies against *T. gondii* tachyzoite antigens, the incubation of western blot strips was performed as previously described for *N. caninum* [15]. In this test, only the reactions against three immunodominant antigens (TG-IDA) of about 20, 30 and 35 kDa were recorded [16]. In the *T. gondii* immunoblot using purified p30 (TgSAG1), the reactivity of sera with a single band of 30 kDa was recorded. In all immunoblots, peroxidase conjugated anti-mouse IgG (H + L) (Jackson Immunoresearch Laboratories, West Grove, PA, USA) was used diluted 1:250 in PBS-TG.

### *Toxoplasma gondii* specific magnetic capture

In order to increase the sensitivity for the detection of the parasite DNA in downstream qPCR reactions, *T. gondii* specific magnetic capture [17] was completed on 100 g tissue pools. The same pooled tissues which were described previously for the mouse bioassay were used for qPCR. In addition, magnetic capture using up to 100 g of remaining non-pooled tissue from heart, liver, diaphragm, *musculus triceps femoralis*, *musculus semitendinosus* and masseter were tested individually.

### *Toxoplasma gondii* specific 529 bp qPCR

The *T. gondii* specific 529 bp qPCR was completed on DNA from magnetic capture samples, DNA extracted from mouse brains and DNA from calf tissue homogenate. All PCR amplifications were performed in 96-well plates using the LightCycler 480 thermal-cycler (Roche Diagnostics Ltd., Burgess Hill, UK) targeting the 529 bp repeat element [18], using the methodology described in [19], but adapted by using 8 μl template DNA and the exclusion of BSA. A qPCR reaction was considered positive if all negative controls and the DNA extraction control were negative with the Cq-value of the sample < 40, and the shape of the amplification curve similar to the positive controls. Samples with a Cq-value < 35 were considered as definitive positives, whilst those with a Cq-value between 35 and 40 were further confirmed by identification of the correct sized band (162 bp) by agarose gel electrophoresis.

## Results

### Rectal temperatures and clinical signs of infection in calves

**Fig. 1:**
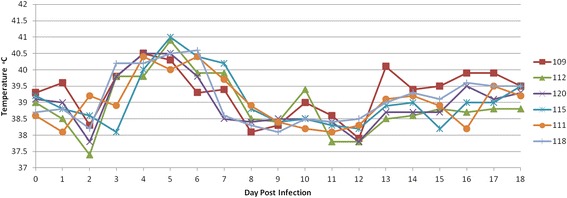
Rectal temperatures of all calves between 1–18 days after oral inoculation with 10^6^ *T. gondii* oocysts (M4 strain). The highest temperature observed was recorded on days 4–6 post-infection and ranged from 40 °C to 41 °C

Following oral infection with 1 × 10^6^ *T. gondii* oocysts, the rectal temperature of all calves began to increase on day 2 pi and peaked on day 5 pi (maximum temperature recorded was 41 °C, calf 115) (Fig. 1). The rectal temperature of calf 109 consistently remained above 39.3 °C from day 13 pi to 18 pi. This calf was suffering from pneumonia (unrelated to the *T. gondii* infection) and had to be excluded from the study on day 40 pi.

### *Toxoplasma gondii* serology in calves as tested by MAT

All calves were seronegative for *T. gondii* by MAT at the start of the experiment (day 0 pi). Following infection an initial immune response was observed by day14 pi, with all animals becoming seropositive by day 21 pi (Table 1).

**Table 1:** Reciprocal titre of *T. gondii* antibodies by MAT for calves orally inoculated with 10^6^ *T. gondii* oocysts (M4 strain). Cut off value ≥ 6 indicates a positive result

Day pi	Calf number					
	109	112	120	115	111	118
0	0	0	0	0	0	0
7	0	0	0	0	0	0
14	50	10	6	100	3	25
21	1600	1600	400	800	800	1600
28	1600	1600	1600	1600	800	1600
35	1600	800	1600	1600	800	1600
42	na	800	800	800	1600	1600

*Abbreviations*: na, animal died due to pneumonia, therefore no sample available for testing; pi, post-infection

### Mouse bioassay, qPCR of tissue homogenate and MC-qPCR of calf tissues

Due to exclusion of calf 109, tissue homogenate from pool 1 consisted of two animals (112 and 120). Within the group of mice which were inoculated with tissue homogenate from pool 1 calves, two mice [46–1 (inoculated with brain) and 45–1 (inoculated with tongue)] were culled due to clinical signs consistent with *T. gondii* infection in mice on days 6 and 8, respectively. However, parasite DNA could not be detected by qPCR from the brains of these two mice or from any other mice inoculated with tissue homogenate from pool 1 calves (Table 2). An additional mouse (46–3) was culled on day 3, however clinical signs were not typical of *T. gondii* infection and both the ELISA results, qPCR on the tissue homogenate and calf tissues were all negative. Following magnetic capture and qPCR, all calf tissues from pool 1 tested negative (Table 2). One positive sample was identified by qPCR from the tissue homogenate in pool 1 (*triceps femoralis* muscle), which showed a very low concentration of *T. gondii* DNA (Cq 37.20), of which the duplicate sample was negative. This qPCR product was examined by gel electrophoresis and a weak band of the correct size was observed.

**Table 2:** Mouse bioassay, qPCR on tissue homogenate, and MC-qPCR detection of *T. gondii* from pooled calf tissues by mouse bioassay (six weeks post-oral inoculation with 10^6^ M4 *T. gondii* oocysts). Figures in bold indicate a strong positive ELISA or real-time PCR (qPCR) result

Calf pool	Tissue	Mouse 1				Mouse 2				Tissue homogenate	Calf tissues
		Mouse No.	Cull date (dpi)	ELISA result (%SP)	qPCR (Cq)	Mouse No.	Cull date (dpi)	ELISA result (%SP)	qPCR (Cq)	qPCR (Cq)	MC-qPCR (Cq)
Pool 1 = ×2 animals (112, 120)	Liver	44-1	42	neg	neg	44-3	42	neg	neg	neg	neg
	Masseter	44-10	42	neg	neg	44-30	42	neg	neg	neg	neg
	Tongue	45-1	8	No sample	neg	45-3	42	neg	neg	neg	neg
	Heart	45-10	42	neg	neg	45-30	42	neg	neg	neg	neg
	Brain	46-1	6	No sample	neg	46-30	3	neg	neg	neg	neg
	Diaphragm	46-10	42	neg	neg	46-30	42	neg	neg	neg	neg
	Left *triceps femoralis* (forelimb)	47-1	42	neg	neg	47-30	42	neg	neg	37.20^a,c^	neg
	Left *semitendinosus* (hindlimb)	47-10	42	neg	neg	47-30	42	neg	neg	neg	neg
	*Psoas major* (fillet)	48-1	42	neg	neg	48-3	42	neg	neg	neg	neg
	*Longissimus dorsi* (sirloin)	48-10	42	neg	neg	48-30	42	neg	neg	neg	neg
Pool 1 = ×3 animals (115, 111, 118)	Liver	53-1	42	neg	**33.79**	53-3^d^	42	neg	33.83^a^	neg	**34.40**
	Masseter	50-10	42	neg	neg	50-30	42	neg	neg	neg	**35.54**
	Tongue	50-1	12	24	**19.66**	50-3	12	neg	**19.04**	**33.37**	**32.34**
	Heart	49-10	42	neg	34.01^a^	49-30	42	neg	**33.53**	neg	**32.84**
	Brain	51-1	12	12	**21.56**	51-3	12	**13**	**21.70**	neg	neg
	Diaphragm	49-1	12	8	**23.09**	49-3	12	**16**	**21.47**	**29.04**	**34.16**
	Left *triceps femoralis* (forelimb)	52-1^d^	42	neg	33.60^a^	52-3	42	neg	neg	neg	**34.63**
	Left *semitendinosus* (hindlimb)	52-10	42	**285** ^**b**^	**18.43**	52-30	42	**230** ^**b**^	**19.87**	**33.92**	**32.54**
	*Psoas major* (fillet)	53-10	42	neg	**18.13**	53-30^d^	42	neg	**19.90**	neg	**34.09**
	*Longissimus dorsi* (sirloin)	51-10	42	neg	neg	51-30	42	neg	neg	neg	neg

Notes on tissue amounts: 50 g per calf (pool 1, as one calf was removed prior to the end of the experiment) and 33.3 g per calf (pool 2); each pool consisted of 100 g

^a^A weak positive ELISA or real-time PCR result; ELISA results are expressed as sample to positive control ratios (%S/P); real-time PCR results are recorded as quantification cycle (Cq)

^b^Between day 10–14 post-inoculation mice showed signs of *T. gondii* infection

^c^Repeated twice and on both occasions one duplicate was negative

^d^Mice positive by total *T. gondii* tachyzoite immunoblot (52–1, 53–30) or positive in p30 (TgSAG1) immunoblot (52–1, 53–3)

*Abbreviation*: neg, negative result

For pool 2 (which consisted of tissues from 3 calves; 115, 111 and 118), mice inoculated with the *semitendinosus* homogenate were both positive for *T. gondii* by ELISA and qPCR. Six mice (50-1, 50-3, 51-1, 51-3, 49-1 and 49-3) from pool two were culled early (day 12) due to clinical signs of *T. gondii* infection*.* These mice had been inoculated with tissue homogenate from tongue, brain and diaphragm and all six mice tested positive by qPCR. In addition, five of these six mice showed a very weak antibody reaction in the ELISA (Table 2). Eight mice from pool 2 (inoculated with homogenate from liver, tongue, heart, triceps and *psoas major*) were also qPCR-positive, but negative by ELISA. The mouse bioassay results correlate well with the MC-qPCR results for calf tissues; the only exceptions are a qPCR positive result in mouse bioassay for brain of pool 2 and a positive MC-qPCR result for masseter for pool 2. MC-qPCR of the different tissue pools resulted in Cq-values varying from 32.34 to 35.54. Only three tissues from pool 2 (tongue, diaphragm and *semitendinosus*) tested positive by qPCR on the inocula (Table 2).

There were eight cases where the ID.Vet ELISA on mouse serum was negative despite *T. gondii* parasite DNA detected from the mouse brain. These eight serum samples (representing mice 49-10, 49-30, 50-3, 52-1, 53-1, 53-3, 53-10 and 53-30), were further tested using the total tachyzoite or the p30 (Tg-SAG1) immunoblot, which resulted in an additional three samples (52-1, 53-3, 53-30) being identified as positive (Table 2).

Testing of individual heart, liver, diaphragm, *triceps femoralis*, *semitendinosus* and masseter samples by MC-qPCR resulted in weak positive reactions of the correct band size for the diaphragm of calf 109 and the heart of calf 112 (Cq values of 36.91 and 37.91, respectively). Positive reactions were obtained from *triceps femoralis*, *semitendinosus* and masseter muscles of calf 118 (Table 3). Other tissues were not tested individually, as following mouse bioassay and pooled tissue MC-qPCR there was not enough material remaining.

**Table 3:** Detection of *T. gondii* by magnetic capture PCR (MC-qPCR). Weight of tissues tested and quantification cycle (Cq) values of DNA extracted from tissues of calves 6 weeks post-oral inoculation with 10^6^ M4 *T. gondii* oocysts. Figures in bold indicate a positive qPCR result. Calf 109 removed prior to the end of the experiment

Calf No.	Calf pool	Tissue											
		Heart		Liver		Diaphragm		Triceps femoralis		Semitendinosus		Masseter	
		Amount for MC (g)	qPCR (Cq)	Amount for MC (g)	qPCR (Cq)	Amount for MC (g)	qPCR (Cq)	Amount for MC (g)	qPCR (Cq)	Amount for MC (g)	qPCR (Cq)	Amount for MC (g)	qPCR (Cq)
109	Excl	82	neg	85	neg	74	**36.91**	91	neg	100	neg	81	neg
112	1	91	**37.91**	100	neg	8	neg	70	neg	100	neg	95	neg
120		77	neg	95	neg	48	neg	55	neg	57	neg	69	neg
115	2	100	neg	100	neg	56	neg	94	neg	94	neg	50	neg
111		100	neg	100	neg	66	neg	100	neg	100	neg	37	neg
118		89	neg	96	neg	na	na	65	**32.27**	98	**32.63**	69	**29.45**

*Abbreviations*: na, insufficient amount of tissue for analysis; neg, negative result

## Discussion

To study the dissemination of *T. gondii* in cattle, six calves were orally inoculated with oocysts. Serological detection of *T. gondii* in calves was performed using MAT, with high titres from all calves at day 21 pi onwards (Table 1), demonstrating that inoculation had resulted in successful infection in all six calves. This was further confirmed by a febrile reaction between 4 and 6 pi within all animals, which is consistent with results observed during other experimental infections with *T. gondii* oocysts in cattle [10, 20, 21] and sheep [22, 23]*.* The serological results are similar to what was observed in a previous *T. gondii* experimental infection of cattle where both IFAT and dye test were used to monitor *T. gondii* titres following experimental infection [21]. Despite all six calves showing seropositivity, *T. gondii* was not detected in all animals. The use of qPCR on the tissue homogenate did not appear to be as sensitive as MC-qPCR on the calf tissues directly or mouse bioassay of tissues, with the only positive samples identified within pool 2 from tongue, diaphragm and left *semitendinosus* by qPCR on the tissue homogenate, in contrast to eight and nine different tissues pools by magnetic capture followed by qPCR and mouse bioassay, respectively. However, parasite DNA was also detected in the left *musculus triceps femoralis* tissue homogenate of pool 1 calves, whereas none of the pooled tissues from these calves tested positive in mouse bioassay or qPCR on tissues following magnetic capture. A lower sensitivity for qPCR relative to bioassay on tissue homogenate is not surprising as the volume inoculated into mice (1 ml of tissue homogenate per mouse) was larger than the volume used for molecular detection (200 μl of tissue homogenate extracted in 100 μl of which only 8 μl of template DNA was used for qPCR).

The mouse bioassay results were based on three criteria; clinical signs of *T. gondii* infection (e.g. stary stiff coat and a hunched appearance), detection of anti-*T. gondii* antibodies in the mice and positive qPCR results for parasite DNA in mouse brains. These results did not always agree with each other. First, on days 6 and 8 pi, two mice (46-1 and 45-1) that received a tissue homogenate from pool 1 had to be euthanised due to clinical signs consistent with *T. gondii* infection, however, it was too early for detection of a specific IgG response and parasite DNA could not be detected from the brains of either of these mice. The mouse culled on day 6 pi received brain homogenate, while the mouse culled on day 8 pi received tongue homogenate. However qPCR of these tissue homogenates and MC-qPCR of the corresponding tissue pools did not identify parasite DNA, meaning it was not possible to make a final conclusion regarding the infection status of these mice and the corresponding calf tissues. The clinical signs observed could have been unrelated to *T. gondii* infection, or the mice may still have been in the acute phase of infection, with tachyzoite multiplication occurring, but too early for the parasite to have reached detectable levels in the brain. Secondly, serological detection of *T. gondii* from mice used in the bioassay (using the ID.Vet ELISA) did not provide an accurate indication as to whether the mice were infected with the parasite (Table 2). Weak ELISA results for mice inoculated with tongue, brain and diaphragm digests can be explained by the need to cull them early, leaving less time for the mouse to develop a strong immune response. However, sera from eight mice which had qPCR-positive brains, but were ELISA negative (49-10, 49-30, 50-3, 52-1, 53-1, 53-3, 53-10, 53-30), were re-tested using the total tachyzoite or the p30 (TgSAG1) immunoblot. The immunoblots identified a further three seropositive mice (52-1, 53-3 and 53-30), resulting in a better agreement between serology, qPCR and bioassay results, as the brains from these mice were also qPCR-positive and the corresponding calf tissues they had been inoculated with were also positive by MC-qPCR. In summary, the mouse bioassay results for pool 2 indicate dissemination of the parasite within different calf tissues: (i) mice inoculated with tongue, brain and diaphragm had to be culled early and had a higher parasite burden, as indicated by a low Cq value; (ii) mice inoculated with *semitendinosus* muscle had a high parasite burden (indicated by a low Cq-values) and strong antibody responses; (iii) mice inoculated with *psoas major* muscle also showed low Cq-values but were negative in ELISA; and (iv) mice inoculated with liver, heart and triceps muscle were ELISA negative and had higher Cq-values but were still classified as qPCR-positive. Mice inoculated with masseter muscle and *longissimus dorsi* muscle remained qPCR and ELISA negative.

Results from previous studies for the detection of *T. gondii* from experimentally infected cattle, have shown that mouse bioassay alone may not be sufficient to detect the presence of *T. gondii* [10, 24], therefore using bioassay in combination with molecular detection of the parasite may increase sensitivity. In this study, detection of parasite DNA by MC-qPCR from pooled tissues agreed well with the mouse bioassay results. When examining the MC-qPCR results from pools of specific tissues, (tongue, *semitendinosus* and heart), these had slightly lower Cq values (ranging from 32.34 to 32.84) than the other MC-qPCR-positive tissues (*psoas major*, diaphragm, liver, triceps and masseter with Cq values ranging from 34.09 to 35.54) (Table 2). In a 100% efficient qPCR, amplified DNA will double with every cycle and 10-fold differences in initial DNA concentration will be 3.3 Cq-values apart [25]. Even the difference in Cq-value between tongue and masseter indicates less than a 10-fold difference in DNA concentration. Based on the current study, it is not possible to identify a single tissue that will provide optimal chances of detecting *T. gondii* when present. It may be better to take several different tissues for testing, such as diaphragm, *semitendinosus* and tongue.

Based on pooled tissues, no conclusion could be made about the infection status of the individual calves, therefore remaining heart, liver, diaphragm, *triceps femoralis*, *semitendinosus* and masseter were tested individually by MC-qPCR. The results show that parasite DNA was present in tissues of the calf that was euthanized early (calf 109: diaphragm) and one calf from pool 1 (calf 112: heart). However, only one of the pool 2 calves (calf 118) tested positive (*triceps femoralis*, *semitendinosus* and masseter muscle). Some discrepancy between positive tissues identified in pooled and individual testing can be expected as different parts of each tissue were cut and used for two separate magnetic captures, in addition, parasite concentration is expected to be low in cattle [2, 26] and only a small percentage of calf tissues were tested. Although it cannot be ruled out that any of the other pool 2 calves harbored *T. gondii* tissue cysts, these results suggest that the positive results for pool 2 are likely attributed to calf 118 alone.

Although no clear predilection site has been identified and there is no correlation between detection of antibodies and detection of tissue cysts, it was not possible to test all calf tissues in the mouse bioassay individually, and with the inhomogeneous nature of tissues cysts, compounded with the limited size of tissue sample which we could collect and test, could mean that cysts may have been missed. Although a cat bioassay would have enabled a greater sample size to be tested, which would have increased the sensitivity, in this study it was not ethically viable to use this model. In the current study, calves were experimentally infected with what is considered a high number of oocysts (10^6^), yet the parasite was detected less frequently from bovine tissues, compared to similar studies in pigs and lambs which had been infected with a lower number of oocysts (10^3^ and 5 × 10^5^, respectively) [13, 22, 23]. Therefore, in relation to *T. gondii* and food safety, the relative risk from undercooked/raw infected beef may be low compared to undercooked/raw infected pork or lamb. It is possible that when the calves were euthanised the majority of animals had already cleared the parasite (despite remaining seropositive), or that the cysts may develop slower over a longer period of time compared to sheep and pigs. If this was the case, then ending the calf experiment 42 days post-infection may have been too early and the results may have been different if the calves had been euthanized later. Another hypothesis would be that cattle are better able to control tachyzoite multiplication and dissemination, resulting in the formation of fewer tissue cysts. A reduction in the detection of parasite DNA from tissues of lambs experimentally infected with *T. gondii* was evident over a six week period [23]; once cattle are infected they may be efficient at reducing the parasite burden over a similar time frame.

## Conclusions

In conclusion, the results from this study demonstrate that viable *T. gondii* and DNA can be detected in several different calf tissues including meat cuts six weeks after oral inoculation with 10^6^ oocysts. There were tissues with more consistent positive results in the mouse bioassay (i.e. tongue, brain, diaphragm, *semitendinosus* and *psoas major* muscle), which could be of importance for future studies examining the presence of *T. gondii* in cattle by mouse bioassay. Interestingly, even within an experimental setting, there was no correlation between presence of antibodies and the detection of infective tissue cysts or *T. gondii* DNA, as all experimentally infected calves were *T. gondii* seropositive by MAT, but not all calves were positive following mouse bioassay or MC-qPCR. This suggests that when it comes to detecting *T. gondii* in calves, one individual test is not enough to tell the whole story.

## Abbreviations

DNA: Deoxyribonucleic acid
ELISA: Enzyme linked immunosorbent assay
MAT: Modified agglutination test
MC-qPCR: Magnetic capture quantitative polymerase chain reaction
qPCR: quantitative polymerase chain reaction
Tg-SAG1: *Toxoplasma gondii* surface antigen 1
